# Distinct tissue-specific requirements for the zebrafish *tbx5* genes during heart, retina and pectoral fin development

**DOI:** 10.1098/rsob.140014

**Published:** 2014-04-23

**Authors:** Aina Pi-Roig, Enrique Martin-Blanco, Carolina Minguillon

**Affiliations:** CSIC-Institut de Biologia Molecular de Barcelona, Department of Developmental Biology, Parc Científic de Barcelona, C/Baldiri Reixac, 10, Barcelona 08028, Spain

**Keywords:** developmental biology, zebrafish, limb, retina, heart, *tbx5*

## Abstract

The transcription factor *Tbx5* is expressed in the developing heart, eyes and anterior appendages. Mutations in human *TBX5* cause Holt–Oram syndrome, a condition characterized by heart and upper limb malformations. *Tbx5*-knockout mouse embryos have severely impaired forelimb and heart morphogenesis from the earliest stages of their development. However, zebrafish embryos with compromised *tbx5* function show a complete absence of pectoral fins, while heart development is disturbed at significantly later developmental stages and eye development remains to be thoroughly analysed. We identified a novel *tbx5* gene in zebrafish—*tbx5b—*that is co-expressed with its paralogue, *tbx5a*, in the developing eye and heart and hypothesized that functional redundancy could be occurring in these organs in embryos with impaired *tbx5a* function. We have now investigated the consequences of *tbx5a* and/or *tbx5b* downregulation in zebrafish to reveal that *tbx5* genes have essential roles in the establishment of cardiac laterality, dorsoventral retina axis organization and pectoral fin development. Our data show that distinct relationships between *tbx5* paralogues are required in a tissue-specific manner to ensure the proper morphogenesis of the three organs in which they are expressed. Furthermore, we uncover a novel role for *tbx5* genes in the establishment of correct heart asymmetry in zebrafish embryos.

## 2. Introduction

*Tbx5* codes for a T-domain containing transcription factor that has been characterized in many vertebrate species, where it is widely expressed during the development of the heart, the eyes and the anterior set of appendages (tetrapod forelimbs and fish pectoral fins) [1–3]. Mutations in human *TBX5* cause Holt–Oram syndrome (HOS; OMIM#142900), an autosomal-dominant ‘heart–hand’ condition characterized by heart and upper limb malformations [4,5]. Owing to its clinical relevance, several *Tbx5* loss-of-function animal models have been developed to assess the role that this gene may play during the development of the vertebrate heart, limbs and eyes. These studies have shown that in mouse *Tbx5*-knockout embryos, both forelimb and heart development is severely impaired from the earliest stages of development [6–8]. Also, mis-expression of *Tbx5* in the ventral retina produces altered projections of retinal ganglion cell (RGC) axons in chick embryos [9], consistent with a fundamental role for *Tbx5* during eye morphogenesis.

The use of zebrafish as a model system has a series of advantages with respect to mouse, mainly their ease of embryo accessibility and the constant development of new techniques such as morpholino (MO) knock-down and transgenesis. Zebrafish pectoral fins are homologous to tetrapod forelimbs, and using genetic and transgenic techniques it has been shown that the molecular mechanisms governing the initial steps of limb/fin bud outgrowth are conserved between tetrapods and teleosts [10]. Hence, zebrafish are commonly used as a model system to study vertebrate limb development. Heart morphogenesis involves the specification and differentiation of cardiac precursors, the integration of precursors into a tube and the remodelling of the embryonic tube to create a fully functional organ [11]. Similar to limb/fin development, similarities between higher vertebrates and zebrafish heart morphogenesis have established zebrafish as a model to study cardiac development and function [12]. Finally, eye formation requires the coordination of a series of morphogenetic events and the regulated expression of several genes that are similarly conserved among vertebrate models [13].

*Tbx5* function has been investigated during zebrafish development using both a MO knock-down approach and the use of a *tbx5a* mutant strain obtained by ENU-induced mutagenesis (*heartstrings* (*hst*) [14]). In zebrafish, and similar to amniote embryos, *tbx5a* is expressed in the heart, pectoral fins and dorsal retina from the earliest stages of their development. However, embryos with compromised *tbx5a* function show a complete absence of pectoral fins, while heart development is disturbed at a relatively late developmental stage. Defects in eye development have not been thoroughly assessed [14,15]. We identified a novel *tbx5* gene in zebrafish—*tbx5b—*that is co-expressed with its paralogue, now referred to as *tbx5a*, in the developing eye and heart fields and that arose during the teleost-specific genome duplication event that took place during evolution. We hypothesized that functional redundancy of *tbx5a* and *tbx5b* in the developing heart would explain the relatively late phenotypes observed during cardiac development in fish embryos with compromised *tbx5a* function [16].

To test our hypothesis, we have now investigated the consequences of *tbx5a* and/or *tbx5b* downregulation during zebrafish development. Our data show that distinct relationships between *tbx5* paralogues are required in a tissue-specific manner to ensure the proper morphogenesis of tissues with conspicuous expression of *tbx5* genes, namely the developing heart, the retina and the pectoral fins. Finally, we also demonstrate that both *tbx5* paralogues are required to direct both asymmetric events the zebrafish heart undergoes (i.e. heart tube jogging first and looping later), thus uncovering a novel and fundamental role for these genes during the establishment of cardiac left–right asymmetry.

## 3. Results and discussion

To understand the unique and/or redundant roles that the *tbx5* paralogues have during the development of the zebrafish heart, pectoral fin and eye fields, we have used MOs against *tbx5a* and *tbx5b* to downregulate their function during embryonic development either individually or in conjunction. Briefly, we used an anti-*tbx5a* MO oligonucleotide for the coding sequence [15], an anti-*tbx5b* oligonucleotide recognizing the 5′ UTR/coding sequence boundary—*tbx5b(UTR)*—as well as an anti-*tbx5b* oligonucleotide for the exon 3/intron 4 boundary (*tbx5b(SP)* MO). First of all, we characterized the functionality of our MOs by generating chimeric mRNAs containing the *tbx5a* or *tbx5b(UTR)* MO-recognition sites fused to enhanced green fluorescent protein (EGFP). Injection of these RNAs (100 pg) with or without their corresponding target MO (3 ng), showed that, indeed, co-injection of our *tbx5a* and *tbx5b(UTR)* MOs caused disappearance of EGFP signal (electronic supplementary material, figure S1a–d′). In addition, and to assess *tbx5b* gene knock-down efficiency, we performed RT-PCR experiments from embryos that had been injected with either a control MO or a *tbx5b(SP)* MO. This showed that an expected 215 bp (spliced) band was obtained in control embryos in contrast to the 791 bp (unspliced) band observed after injection of 2–4 ng of *tbx5b(SP)* MO (electronic supplementary material, figure S1e).

### 3.1. Knock-down of zebrafish *tbx5* genes causes cardiac looping defects

**Figure 1.**
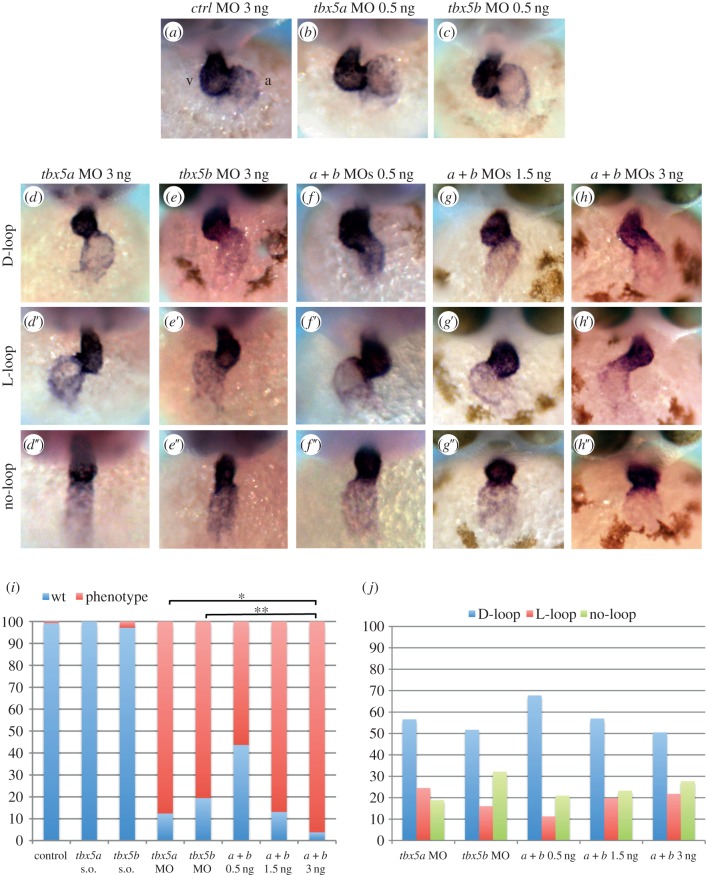
Knock-down of *tbx5* genes causes cardiac looping defects. (*a*–*c*) Embryos injected with control MO or sub-optimal concentrations of *tbx5a* or *tbx5b* MOs. (*d*–*d″*) *tbx5a* morphant phenotypes. (*e*–*e*′) *tbx5b-*morphant phenotypes. (*f*–*h″*) Double knock-down of *tbx5* genes (0.5 ng each MO (*f*–*f″*), 1.5 ng each MO (*g*–*g″*) and 3 ng each MO (*h*–*h″*)). (*i*) Quantification of the degree of looping phenotypes: wt, complete; phenotype, incomplete looping s.o., sub-otimal. (*j*) Quantification of the looping orientation phenotypes. A *χ*^2^ statistic has been calculated to assess significant differences between groups (**p < 0.001, *p < 0.05). Images are frontal views of 48 hpf embryos, and *myl7* expression is used to highlight the developing heart.

*tbx5a* and, as described recently, *tbx5b* have been implicated in cardiac looping morphogenesis [14,17]. Analyses of looping phenotypes assayed at 48 h post-fertilization (hpf) had shown that wild-type embryos had undergone complete looping (i.e. the ventricle located at the right-hand side of the embryo with the atrial and ventricular chambers sitting side by side), whereas homozygous *tbx5a* mutant (*hst*) and *tbx5b* morphants failed to do so, indicating that downregulation of *tbx5* genes was associated with reduced looping. As *tbx5b* knock-down on *hst* mutant embryos did not increase the severity of the defects, it was argued that these paralogues do not have an overlapping function in cardiac development. In addition, looping of these hearts in the reverse orientation was never observed [17]. Similarly, we observed incomplete looping after *tbx5a* or *tbx5b* knock-down (figure 1*d*,*e*, respectively) in comparison to the characteristic looping observed in control morphants that results in the positioning of the ventricle (v) and the atrium (a) parallel to each other (figure 1*a*). However, in striking contrast to previous data, defects were observed not only in the degree of looping but also in the orientation of cardiac looping: *tbx5* morphants can be classified into three distinguishable heart looping orientation groups (D-loop (right, normal), L-loop (left, reversed) and no-loop). After injection of a control MO, over 99% (*n* = 158) of the embryos displayed an S-shaped heart with the ventricle lying to the right-hand side of the embryo (D-loop; figure 1*a*,*i*). By contrast, 88% (*n* = 121) of the *tbx5a* MO-injected embryos had incomplete cardiac looping, and within these 57% showed D-loop, 24% showed L-loop and 19% showed no looping at all (figure 1*d*–*d″*,*i*,*j*). Similarly, injection of *tbx5b* MO also caused heart looping defects (81%, *n* = 108), and these embryos displayed D-looped (52%), L-looped (16%) and no-looped (34%) cardiac morphologies (figure 1*e*–*e″*,*i*,*j*).

The phenotypic similarities observed after *tbx5a* or *tbx5b* knock-down prompted us to investigate whether these genes cooperatively regulate cardiac looping in contrast to what had been previously argued. To this end, we simultaneously knocked-down their function by co-injecting sub-optimal doses of *tbx5a* and *tbx5b* MOs (0.5 ng of each MO) which, when injected alone, did not affect cardiac looping (figure 1*b*,*c*,*i*; *n* = 75 for *tbx5a* MO and *n* = 71 for *tbx5b* MO). This showed that over 56% (*n* = 110) of the double-morphants had looping defects and the three D-loop, L-loop and no-loop phenotypes were detected (68%, 11% and 21%, respectively; figure 1*f*–*f″*,*i*,*j*) suggesting that, indeed, both genes act in the same pathway and cooperate with each other to ensure the completeness and orientation of looping of the zebrafish heart. Moreover, injection of increasing concentrations of both MOs caused an increase in the percentage of phenotypes, with 87% (*n* = 99) and 96% (*n* = 105) of double-morphants displaying looping phenotypes after injection with 1.5 and 3 ng of each MO, respectively. Although, in agreement with a previous report [17], the severity of the phenotype was not enhanced by double knock-down, downregulation of both genes increased the penetrance of the phenotype (figure 1*i*). Similarly to single and double-morphants injected with sub-optimal doses of the *tbx5a* and *tbx5b* MOs, these double-morphant embryos also exhibited looping orientation defects since the three orientation phenotypes were detected (figure 1*g*–*g″*,*h*–*h″*,*j*).

### 3.2. *tbx5* morphants exhibit cardiac tube jogging defects

**Figure 2.**
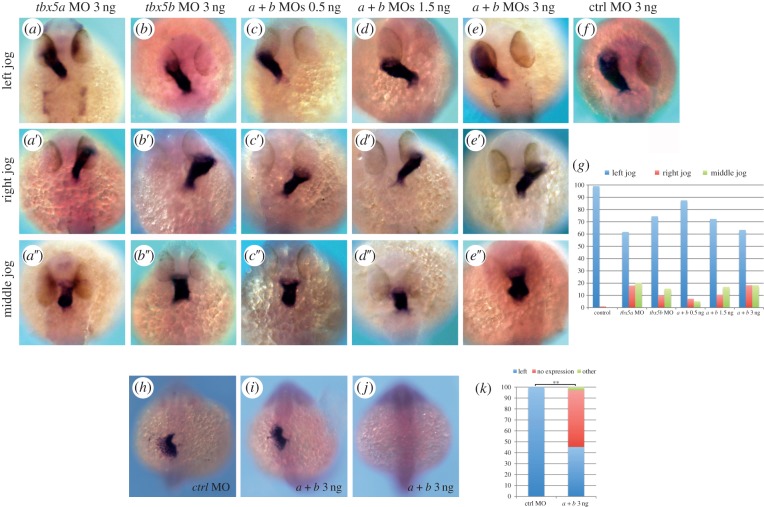
*tbx5* morphants exhibit cardiac jogging and *lefty2* expression defects. (*a*–*a″*) *tbx5a* morphant phenotypes. (*b*–*b″*) *tbx5b* morphants. (*c*–*e″*) Left, right and middle jog phenotypes obtained by co-injection of *tbx5a* and *tbx5b* MOs at 0.5 ng (*c*–*c″*), 1.5 ng (*d*–*d″*) or 3 ng (*e*–*e″*) of each MO. (*f*) Control (ctrl) morphant. (*g*) Quantification of the phenotypes. (*h*–*j*) *lefty2* expression in control (*h*) and double-morphant (*i*,*j*) 22-somite stage embryos. (*k*) Quantification of the phenotypes. A *χ*^2^ test has been used to assess significant differences between groups (**p < 0.001). All images are dorsal views with anterior to the top, and *myl7* expression is used to highlight the developing heart tube in *a*–*f*.

As heart looping orientation phenotypes are indicative of cardiac left–right asymmetry defects, we decided to examine whether heart jogging, the first morphologically evident break in the left–right symmetry of the zebrafish heart tube, was disrupted after knock-down of *tbx5* genes. To this end, heart laterality was assessed at 26 hpf using *myl7* expression to reveal positioning of the heart tube and cardiac jogging was classified as left (normal), right (reversed) or midline (no jog). After injection of 3 ng of control MO, 99% (*n* = 197) of the embryos developed normal left jogging (figure 2*f*,*g*). By contrast, right as well as midline jogging phenotypes were observed after *tbx5a* knock-down (figure 2*a*–*a″*,*g*; *n* = 183). Similarly, *tbx5b* morphants displayed left as well as right and middle jogging of their linear heart tubes (figure 2*b*–*b″*,*g*; *n* = 90).

Once again, the phenotypic similarities between *tbx5a* and *tbx5b* morphants suggested these genes cooperatively regulate heart jogging. To confirm this, we injected sub-optimal doses of *tbx5a* and *tbx5b* MOs which showed that, in agreement with both genes cooperating to ensure the normal left-sided jog of the heart tube, 12.5% (*n* = 96) of these morphant embryos had a defective middle or right jog of the heart (figure 2*c*–*c″*,*g*). Moreover, and similar to the heart looping defects discussed above, injection of increasing concentrations of both MOs increased the percentage of phenotypes observed, with nearly 30% (*n* = 94) and 40% (*n* = 71) of double-morphant embryos showing defective heart jogging after injection of 1.5 and 3 ng of each MO, respectively (figure 2*d*–*e″*,*g*).

The cardiac phenotypes caused by *tbx5a* and/or *tbx5b* knock-down (namely cardiac jogging and looping orientation defects) demonstrate that *tbx5* genes are required to direct both asymmetric events the zebrafish heart undergoes [18]. First, and during the process referred to as cardiac jogging, the cardiac cone, formed at the embryonic midline, is converted into a linear tube by the repositioning of the atrial cells to the anterior and left of the ventricular cells due to their higher migration rates [19,20]. Second, and during the conserved process of cardiac looping, the ventricle is positioned to the right of the atrium. These two processes are defective in *tbx5a* and/or *tbx5b* morphants. Interestingly, known left–right cardiac determinants such as *Bmp4* have been isolated in screens aimed to find *Tbx5*-induced genes [21], and bioinformatic approaches have highlighted the presence of *Tbx5*-binding sites in the vicinity of the *lefty2* locus (N. Mercader 2013, personal communication), another well-known left–right asymmetry determination factor. To address whether *tbx5* genes may be regulating *lefty2* expression in developing embryos, we assessed the expression of this gene after *tbx5* genes knock-down. In support of *tbx5* genes being upstream of *lefty2* expression, over half of the *tbx5a* and *tbx5b* double-morphants (52%, *n* = 106) showed no expression of *lefty2*, whereas 100% of control MO injected embryos (*n* = 43) had the characteristic left-sided expression of *lefty2* at the 22-somite stage (figure 2*h*–*k*). These experiments show that *tbx5* genes are upstream of *lefty2* expression, and hence the cardiac laterality phenotypes observed in *tbx5* morphants may be explained by this relationship between *tbx5* and the asymmetric gene *lefty2*. However, how bilaterally expressed genes such as *tbx5* can specifically regulate a left-side specific gene like *lefty2* remains unclear. Nevertheless, one can hypothesize that *lefty2* will only become activated in the left-hand side of the cardiac cone where *tbx5* acts with a co-activator that is only present in the left side of the developing embryo. Conversely, a repressor only found in the right side of the embryo may be inhibiting the activation of *lefty2* by *tbx5* genes in the right side of the cardiac cone.

**Figure 3.**
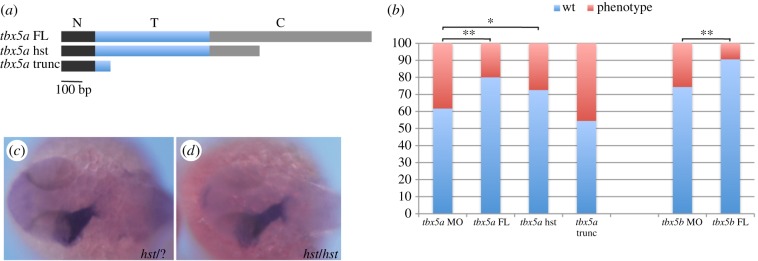
The *hst* mutation is a hypomorphic allele with regards to cardiac laterality. (*a*) *tbx5a* variants generated to perform the rescue experiments: a *tbx5a* full-length (*tbx5a* FL) version includes the whole N-terminal (N, black rectangle), T- (T, blue rectangle) and C-terminal (C, grey rectangle) domains; a heartstrings version (*tbx5a* hst) containing the whole N-terminal domain and T-domain and a truncated C-terminal domain; and a *tbx5a* severely truncated version (*tbx5a* trunc) that contains the whole N-terminal domain and a truncated T-domain. (*b*) Quantification of the rescue experiments (wt, left jog; phenotype, right and middle jog). A *χ*^2^ statistic has been calculated to assess significant differences between groups (**p < 0.01, *p < 0.05). (*c*,*d*) Dorsal views with anterior towards the left of 26 hpf +/+ or +/*hst* (*c*) and *hst* mutant (*d*) embryos showing normal leftward jogging of the embryonic heart tube highlighted by *myl7* expression.

The reasons for the discrepancies between our results, implicating the *tbx5* genes in the asymmetry events the zebrafish heart undergoes (namely cardiac jogging first and looping later), and those of others [17] are unclear. One possibility is that we have used a MO against *tbx5a* to downregulate its function, whereas Parrie *et al*. [17] used the *tbx5a* mutant line *hst* to analyse the effects of this gene loss-of-function. The *hst* mutation introduces a premature STOP codon at residue 316 of the *tbx5a* open reading frame, which leaves the mutated protein with intact N-terminal and T-box (DNA binding) domains and part of the C-terminal domain. It is therefore possible that the *hst* mutation behaves as an hypomorphic allele with regard to the left–right asymmetric development of the heart. In agreement with this hypothesis, most of the *TBX5* mutations causing a clear HOS phenotype lie upstream of the predicted *hst* mutation. To test whether, indeed, the *hst* mutation behaves as an hypomorphic allele with regards to cardiac development, we developed an assay to assess whether the laterality phenotype of *tbx5a* morphants could be rescued by introducing specific MO-insensitive forms of the *tbx5a* mRNA: (i) a full-length *tbx5a* mRNA that should be able to rescue the *tbx5a* MO-mediated phenotype, (ii) a *tbx5a* mRNA that is identical to that produced in *hst* mutant embryos and (iii) a severely truncated version of *tbx5a* that we have engineered by introducing a premature STOP codon within the T-box domain (figure 3*a*). Notably, both the full-length and the *hst*-like forms of *tbx5a* were able to partially rescue the cardiac jogging phenotype when co-injected with the *tbx5a* MO (figure 3*b*; *n* = 65 and *n* = 168, respectively). Similarly, a full-length *tbx5b* form was able to rescue the jogging phenotype of *tbx5b* morphants when co-injected with our *tbx5b* MO (figure 3*b*; *n* = 117). By contrast, the severely truncated form of *tbx5a* was not able to rescue the laterality phenotype (figure 3*b*, *n* = 79). Overall, these data demonstrate not only the specificity of the cardiac phenotypes caused by MO-mediated knock-down of *tbx5a* and/or *tbx5b*, but also that the *hst* mutation behaves as a hypomorphic allele with regard to cardiac laterality. We have ourselves analysed heart tube jogging in *hst* mutants (*n* = 38) and all of them displayed a normal left-jog as visualized by *myl7* expression in 26 hpf embryos (figure 3*c*,*d*). These results underline the need for caution when using the *hst* mutation as a *tbx5a* loss-of-function allele.

### 3.3. *tbx5* genes are essential for correct dorsoventral retina regionalization

*Tbx5* genes are also conspicuously expressed in the dorsal retina, a feature conserved among vertebrates [1–3,22]. However, the consequences of *Tbx5* loss-of-function in this domain have led to controversial results. *Tbx5* gene function in the developing retina has been examined in developing chick embryos by mis-expressing this gene in the ventral domain, where it is not normally expressed. This caused dorsalization of the ventral retina as determined by upregulation of dorsal markers and downregulation of ventral ones, as well as altered projections of RGCs [9]. *tbx5a* knock-down in zebrafish led to downregulation of dorsal retina markers, while ventral markers did not seem to be affected and ganglion cell projections were not analysed [23]. We hypothesized that as *tbx5a* and *tbx5b* are co-expressed in the dorsal domain of the developing retina, functional redundancy may explain the controversial results found between different models. This prompted us to determine the consequences of the downregulation of *tbx5* genes in this territory.

**Figure 4.**
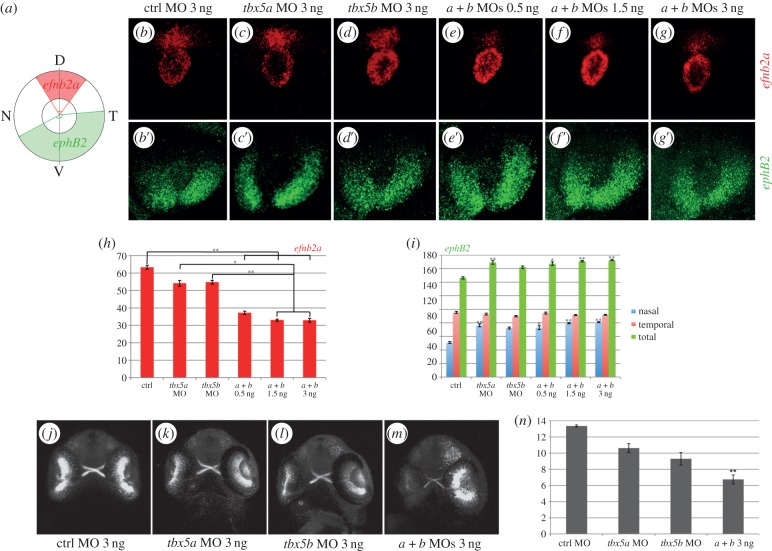
*tbx5* genes are required for dorsoventral retina organization. (*a*) Schematics of our quantification method. Expression of *efn2a* and *ephB2* in control (ctrl) embryos (*b*–*b*′), *tbx5a* morphants (*c*–*c*′) and *tbx5b* morphants (*d*–*d*′). (*e*–*g*′) Expression of *efn2a* and *ephB2* in embryos co-injected with different concentrations of both *tbx5a* and *tbx5b* MOs. (*h*–*i*) Quantification of the results obtained for the expression of *efn2a* (*h*) and *ephB2* (*i*). (*j*–*m*) Retinal projections of 48 hpf *ath5:GFP* embryos injected with control, *tbx5a*, *tbx5b* or *tbx5a* and *tbx5b* MO. (*n*) Optic nerve diameter quantifications. Data are represented as the mean ± s.e. A Kruskal–Wallis test was used to determine statistical differences among experimental groups (*p < 0.05, **p < 0.001). D, dorsal; N, nasal; T, temporal; V, ventral.

We analysed the expression of the dorsally expressed ephrin, *efnb2a*, and the ventrally expressed ephrin receptor, *ephB2*, because restricted ephrinB/ephB expression along the dorsoventral axis has been shown to play a key role in retinotectal topographic map formation [24,25]. To quantify the extent of *efnb2a* expression, we measured the angle of expression of this gene by setting a ‘hinge’ in the centre of the lens (figure 4*a*). In control embryos, the *efnb2a* expression domain was measured to form an average angle of 63° (figure 4*b*,*h*). Knock-down of either *tbx5a* or *tbx5b* caused a reduction of the *efnb2a* angle of expression leading to an average angle of 54°, although this decrease was not found to be statistically significant (figure 4*c*,*d*,*h*). To investigate whether both paralogues function in conjunction to determine the extent of dorsal *efnb2a* expression, we co-injected sub-optimal doses of *tbx5a* and *tbx5b* MOs. Remarkably, the *efnb2a* expression domain was greatly reduced to an average angle of 37°, a statistically significant 41% reduction compared with control embryos (figure 4*e*,*h*). Injection of increasing concentrations of both MOs caused slightly more severe effects than those observed after co-injection of sub-optimal doses of *tbx5a* and *tbx5b* MOs (figure 4*f*,*g*,*h*). Notably, further statistical analyses showed that significant differences are found between the injection of single *tbx5a* and *tbx5b* MOs with respect to their co-injection at these higher doses (1.5 and 3 ng each; figure 4*h*), suggesting that *tbx5* paralogues act redundantly in the dorsal retina to ensure *efnb2a* expression in this territory. To better map the decrease of *efnb2a* expression observed in *tbx5a*- and/or *tbx5b*-morphant retinas, we divided the angles obtained into two, a dorso-nasal angle and a dorso-temporal angle, by setting the dorsal-most point as the point lying dorsal to the ventrally located choroid fissure (D in figure 4*a*). This showed that both dorso-nasal and dorso-temporal borders of expression similarly decreased accordingly to the total angle of *efnb2a* expression measured (electronic supplementary material, figure S2*a*–*c*).

To ascertain whether this decrease in *efnb2a* expression domain was concomitant with a deregulation of *ephB* expression in the ventral retina, we measured the extent of *ephB2* expression. In control embryos, the expression domain of *ephB2* formed an average angle of 146° (figure 4*b*′,*i*). *tbx5a* and *tbx5b* downregulation caused an increase of this angle to an average of 169° and 162°, respectively (figure 4*c*′–*d*′,*i*). Moreover, injection of sub-optimal doses of *tbx5a* and *tbx5b* MOs similarly caused an increase in the expression extent of the ventral marker *ephB2* (to an average of 167°, figure 4*e*′,*i*). Again, injection of increasing concentrations of both MOs caused similar effects to those observed after co-injection of sub-optimal doses of *tbx5a* and *tbx5b* MOs (figure 4*f*′–*g*′,*i*). In contrast to the results obtained for the bilaterally symmetrical loss of expression of the dorsal marker *efnb2a*, statistically significant expansions of the *ephB2* expression domain towards its ventro-nasal border were largely enough to explain the increase in the global domain of *ephB2* expression in the different conditions (figure 4*i*).

Interestingly, the homeodomain transcription factor *meis1* has been implicated in the establishment of the proper retinotectal map of the developing zebrafish. *meis1* knock-down causes a decrease of dorsal *efnb2a* expression and an increase of ventral *ephB2*, which is also associated with downregulation of dorsal *tbx5a* expression [26]. We show that downregulation of *tbx5a* is enough to explain the defects in dorsoventral-restricted expression of the ephrinB/ephB and therefore propose a model by which *meis1* acts upstream of *tbx5* genes expression to ensure the correct dorsoventral expression of *efnb2a* and *ephB2* in the developing retina.

Notably, dorsal *efnb2a* gene expression is not completely abolished after *tbx5a* and *tbx5b* knock-down, suggesting that other factors are acting with the *tbx5* paralogues to maintain dorsal retina identity. Strikingly, T-box genes have been shown to cooperatively interact in many developmental processes [27,28]. Regarding dorsal retina identity, several related T-box genes are co-expressed with *tbx5* in this domain, namely the other three genes that, together with *tbx5*, form the *Tbx2* subfamily of T-box genes, i.e. *tbx2*, *tbx3* and *tbx4* [29–31]. Functional redundancy between these genes may therefore explain the lack of complete downregulation of dorsal retina markers.

Finally, to assess whether altered *efnb2a* and *ephB2* expression in *tbx5a* and/or *tbx5b* morphants altered the normal formation of the retinotectal map, we injected our MOs into one-cell stage *ath5*:*gfp* embryos that express the *gfp* transgene in RGCs under the regulation of the *ath5* promoter, the zebrafish homologue of the *Drosophila atonal* gene [32]. By 48 hpf, RGCs have extended their axons to form the optic nerve that crosses the ventral midline to form the optic chiasm and project dorsally to the contralateral optic tectum (figure 4*j*). Injection of *tbx5a* and/or *tbx5b* MOs did not cause observable pathfinding errors (figure 4*k*–*m*). However, these experiments showed that the optic nerve of double-morphants was considerably thinner than that of control MO-injected siblings (figure 4*m*). Moreover, and in agreement with both *tbx5* genes acting redundantly to ensure proper optic nerve formation, double-morphant embryos showed a significantly thinner optic nerve (figure 4*n*). It is noteworthy that *space cadet* mutants, fish that carry a mutation in the retinoblastoma gene *rb1*, exhibit thinner optic nerves than wild-type siblings [33] and that it has been shown that Tbx2 (closely related to Tbx5) molecularly interacts with Rb1 [34]. It is therefore tempting to speculate that, likewise, Tbx5 may be interacting with Rb1 to regulate the normal formation of the optic nerve in zebrafish embryos.

Altogether, our data show that knock-down of *tbx5* genes causes an alteration of dorsoventral *ephrinb*/*ephB* expression in the retina and the formation of a thinner optic nerve.

### 3.4. *tbx5b* knock-down causes a delay in pectoral fin growth

**Figure 5.**
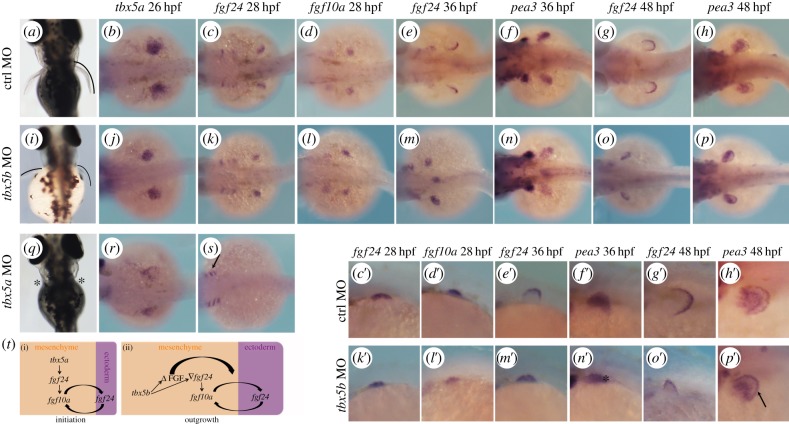
*tbx5b* knock-down causes a delay in pectoral fin growth. (*a*,*i*,*q*) Pectoral fin morphology at 3 dpf. Dorsal views are shown with anterior to the top. (*b*–*h*) Expression of the developing pectoral fin markers in control (ctrl) MO-injected embryos. (*c*′–*h*′) Higher magnifications of (*c*–*h*). (*j*–*p*) Pectoral fin markers expression in *tbx5b*-morphant embryos. (*k*′–*p*′) Higher magnifications of (*k*–*p*). (*r*,*s*) *tbx5a* morphants. (*t*) Model for the differential requirements for the *tbx5* genes during pectoral fin development. *b*–*h*, *j*–*p*,*r*,*s* are dorsal views with anterior to the left. *c*′–*h*′,*k*′–*p*′ are lateral views with anterior to the left.

Although we had not previously observed *tbx5b* expression in developing pectoral fins [16], others have recently described it in the pectoral fin bud mesenchyme of 36 hpf embryos [17]. In agreement with *tbx5b* playing a role during zebrafish pectoral fin morphogenesis, *tbx5b* morphants had smaller pectoral fins when compared with control embryos at 3 dpf (figure 5*a*,*i*; [17]), which is reminiscent of the phenotypes observed upon subtle downregulation of *tbx5a* function [14,15] or downregulation of *tbx5* target genes [35]. To get further insight into where in the limb developmental pathway *tbx5b* function is required, we used a series of markers to assess the state of the two tissues required for and involved in the process of fin outgrowth: the fin mesenchyme and the overlying fin ectoderm. Briefly, bi-directional fibroblast growth factor (FGF) signals emanate from and are received by both tissues, creating a positive feedback loop that is required to sustain pectoral fin outgrowth [10].

In control embryos, compacted pectoral fin territories can be observed by means of *tbx5a* expression at 26 hpf (figure 5*b*) and expression of the *tbx5a* target gene, *fgf24*, is activated in this territory, namely the pectoral fin mesenchyme (figure 5*c*–*c*′). By contrast, *tbx5a* morphants failed to compact the *tbx5a*-labelled mesenchymal tissue (figure 5*r*) and *fgf24* expression was never activated in the mesenchyme although its expression was readily detected in other tissues such as the branchial arches (figure 5*s*, arrow). Similar to control embryos, *tbx5b* morphants displayed compacted expression of *tbx5a* and *fgf24* expression was activated in this territory (figure 5*j*,*k*–*k*′). *fgf24* expressed in the pectoral fin bud mesenchyme is required to activate the expression of another FGF, namely *fgf10a* [36]. Activation of *fgf10a* expression is observed in both control and *tbx5b* morphants (figure 5*d*–*d*′,*l*–*l*′), indicating that the fin bud outgrowth initiation programme is properly established in *tbx5b*-compromised embryos. However, in contrast to the similarities observed between control and *tbx5b* morphants regarding mesenchymal FGF expression, it was striking to note that at 36 hpf, when *fgf24* expression has been downregulated in this tissue and activated in the overlying fin ectoderm in control embryos, this did not occur after *tbx5b* depletion (figure 5*e*–*e*′,*m*–*m*′). To determine whether, indeed, the only tissue with active FGF signalling was the fin bud mesenchyme and not the overlying ectoderm of *tbx5b* morphants, we used *pea3* expression, a direct read-out of cellular exposure to FGF, as a marker [37]. In control embryos, *pea3* expression is detected in both mesenchymal and ectodermal compartments of the fin bud (figure 5*f*–*f*′), whereas only mesenchymal *pea3* was detected after *tbx5b* knock-down (figure 5*n*–*n*′; the asterisk labels lack of ectodermal expression). Interestingly, ectodermal *fgf24* was observed in 48 hpf *tbx5b*-morphant fins (figure 5*o*–*o*′) and these fins resembled control 36 hpf ones (figure 5*e*′,*o*′). In agreement with FGF signalling being active in both mesenchymal and ectodermal tissues of *tbx5b*-depleted fins, *pea3* expression was now evident in these two tissues (figure 5*p*–*p*′; the arrow marks expression in the *tbx5b*-morphant ectoderm), similarly to what is found in control MO-injected embryos (figure 5*h*–*h*′).

Taken together, we show that pectoral fin development has a different requirement for each of the *tbx5* paralogues: *tbx5a* function is required for the earliest steps of initiation of fin outgrowth, whereas *tbx5b* functions later to ensure properly timed and sustained fin outgrowth. Both these requirements are linked by the connection between *tbx5* genes and the downstream regulation of FGF signalling. Briefly, during the initiation of pectoral fin outgrowth (figure 5*t*(i)), *tbx5a* expressed prior to overt fin outgrowth is required to initiate *fgf24* signalling in the pectoral fin mesenchyme, and hence in the absence of *tbx5a* the limb initiation programme is never established and pectoral fins fail to form (figure 5*q*, asterisks; [14,15]). Later, once the limb initiation programme has commenced and FGF signalling is active in the pectoral fin mesenchyme, *tbx5b* is required for the maintenance of pectoral fin growth. *tbx5b* morphants have a delay in pectoral fin growth: these embryos fail to downregulate *fgf24* expression in the fin bud mesenchyme and activate this gene expression in the overlaying ectoderm at 36 hpf. Nevertheless, 12 h later, *fgf24* expression is no longer detected in the mesenchyme and becomes clearly observed in the ectodermal tissue. Strikingly, 48 hpf *tbx5b*-morphant pectoral fins resemble younger (36 hpf) control fins, suggesting that *tbx5b* is required to setup a certain threshold of FGF activity in the mesenchyme that is necessary to (i) signal to the overlying ectoderm and activate FGF signalling in this tissue and (ii) downregulate *fgf24* expression in the mesenchyme (figure 5*t*(ii)). Thus, owing to this delay in FGF signalling activation in the ectoderm, *tbx5b*-deficient pectoral fins appear smaller than control fins (figure 5*a*,*i*). Given the critical requirement of *tbx5a* to establish the pectoral fin bud outgrowth initiation programme, it is not clear whether *tbx5a* may function, similarly to *tbx5b*, during these later stages of fin outgrowth. It is tempting to speculate that this is indeed the case, because, as mentioned before, the subtle downregulation of *tbx5a* function or its target genes is reminiscent of the *tbx5b* loss-of-function shown here.

### 3.5. Distinct tissue-specific requirements for *tbx5a* and *tbx5b*

Our characterization of the consequences of *tbx5a* and/or *tbx5b* knock-down in the three tissues where these genes are expressed demonstrates the existence of differential requirements for these paralogues in each tissue and distinct functional inter-relationships between the *tbx5* genes.

Firstly, cardiac looping is affected in both single *tbx5a* and *tbx5b* morphants, and downregulation of both *tbx5* genes does not increase the severity of the looping phenotype, indicating the essential function each of these genes plays to achieve the complete looping of the zebrafish heart. Moreover, we show that both genes act in the same pathway and cooperate with each other to ensure looping morphogenesis, because co-injection of sub-optimal concentrations of *tbx5a* and *tbx5b* MOs similarly causes looping phenotypes and increasing concentrations of both MOs caused an increase in the percentage of phenotypes (figure 1*i*). With regard to heart asymmetric development, we also show that both paralogues are essential for normal leftward heart tube jogging and consequent dextral looping to occur, because *tbx5a* and *tbx5b* morphants show jogging and looping orientation defects and co-inhibition of both genes does not result in either more severe or higher phenotypic penetrance (figures 1*j* and 2*g*). Secondly, a synergistic effect of *tbx5a* and *tbx5b* is necessary for proper *efnb2a* expression in the dorsal retina. *efnb2a* expression is not significantly affected in *tbx5a* or *tbx5b* morphants, whereas it is decreased by 50% in double *tbx5a* and *tbx5b* morphants, suggesting these two genes act together to guarantee the proper extent of *efnb2a* expression in this domain. Further, the optic nerves of double-morphants appear thinner than those of control siblings (figure 4). Finally, it is noteworthy that still another relationship between *tbx5* genes is found regarding fin bud development, where *tbx5a* and *tbx5b* are differentially required to ensure the proper initiation of outgrowth first and maintenance of fin growth later, respectively (figure 5).

The tight regulation of *Tbx5* gene dosage has been shown to be fundamental for many developmental processes to take place normally, because both the subtle upregulation and downregulation of its function has been shown to cause developmental defects [38–41]. Our data demonstrate that, moreover, *tbx5* gene(s) dosage needs to be strongly controlled in a tissue-dependent manner to ensure the proper morphogenesis of the distinct tissues where this gene is most prominently expressed.

## 4. Material and methods

### 4.1. Animal welfare

The local ethics committee approved animal studies and all procedures conformed to the essential ethical rules and the current applicable legislation (Council Directive 86/609/EEC; Law 5/1995/GC; Order 214/1997/GC; Law 1201/2005/SG). Adult fish are kept in a designated fish facility with a designated manager and welfare officers. When animals need to be euthanized, an overdose of tricaine methane sulfonate (MS222, 200–300 mg l^−1^) by prolonged immersion was used, which is a well-established humane method.

### 4.2. Animal maintenance

Adult zebrafish were bred under standard conditions and embryos obtained by natural spawning and incubated at 28.5°C in E3 medium [42]. They were further staged and fixed at specific time-points as described by Kimmel *et al.* [43]. Wild-type and *ath5*:*GFP* [32] fish were used in this study.

### 4.3. Morpholino oligonucleotides

MO oligonucleotides (Gene Tools LLC) were dissolved to 1 mM and 0.5–3 ng injected into one-cell stage embryos. MOs were co-injected with an anti-*p53* MO (7.5 ng) to avoid off-target effects caused by toxicity, and all experiments were performed with at least three independent replicates. The MOs used were: a control MO; an anti-*tbx5a* MO for the coding sequence [15]; an anti-*tbx5b* MO against the 5′UTR/coding sequence boundary—*tbx5b(UTR)* MO—5′ GGATTCGCCATATTCCCGTCTGAGT 3′; and an anti-*tbx5b* oligonucleotide for the exon 3/intron 4 boundary—*tbx5b(SP)* MO—5′ TTAAAAAACTAGGCACTCACCGGCC 3′. To test the knock-down efficiency of the *tbx5b(SP)* MO, RT-PCR was performed using whole-embryo RNA from 24 hpf embryos that had been injected with either control or *tbx5b(SP)* MO. RNA was isolated using Trizol reagent (Invitrogen) and a reverse transcription reaction with SuperScript II RNase H—reverse transcriptase (Invitrogen) was then performed to generate cDNA following the manufacturer's instructions. The PCR was performed using the primers *zftbx5b_ex3fwd* 5′ AGTATGGAGGGAATTAAAGTTTA 3′ (sequence present in the third exon of the *tbx5b* gene) and *zftbx5b_ex4rev* 5′ CATTTGTTATCTGCAAACTTATAC 3′ (present in the fourth exon of the *tbx5b* gene) to detect spliced and un-spliced *tbx5b* transcripts. As equivalent phenotypes were obtained with both *tbx5b* MOs, for most of the experiments results with the *tbx5b(UTR)* MO are shown, unless otherwise indicated.

### 4.4. Morpholino functionality and specificity

To assess for the functionality of the MOs used, we generated chimeric mRNAs in which the *tbx5a* or *tbx5b(UTR)* MO recognition sites (underlined) were fused to EGFP by PCR amplification using a plasmid containing *EGFP-polyA* as template. The following primers were used: *tbx5aMO_EGFP_fwd*, 5′ ATGGCGGACAGTGAAGACACCTTTCGGGTGAGCAAGGGCGAGGAGC 3′; *tbx5bMO_EGFP_fwd*, 5′ ACTCAGACGGGAATATGGCGAATCCAGTGAGCAAGGGCGAGGAGC 3′; in conjunction with the reverse primer: *FP_SV40rev* 5′ AAGCTTGATGAGTTTGGACAAACCAC 3′. The resulting products were cloned into the pGEM T-easy vector (Promega) and further transferred into the *pCS2+* vector to obtain full-length mRNAs. The mMessage Machine kit (Ambion) was used to obtain full-length mRNAs according to the manufacturer's protocol. One hundred picograms of mRNA with or without the corresponding MO (3 ng) was injected into one-cell stage embryos and the presence of GFP expression assessed at 24 hpf.

To assess for the specificity of the MOs used and functionality of *tbx5a* variants, mRNAs to perform rescue experiments were generated. The following primers were used (mis-matched nucleotides are shown in small letters): *tbx5a_fwd*, 5′ ATGGCcGAttcaGAgGAtACgTTcaGGCTCCAAAACTCTCCCAGTG 3′ in conjunction with *FLrev* 5′ TTAGCTGGCTTCATTCCAGTC 3′, *hstQ316rev* 5′ CTaTGTGTGTCCGTGGTAGGAGC 3′ or *T-boxtruncrev* 5′ CTATGCTTTGGTGATGATCATCTCTG 3′ were used to generate full-length, heartstrings or truncated variants of tbx5a, respectively. *tbx5b_fwd* 5′ ATGGCcAAcCCAATGTTCGAATCTCTACGG 3′ and *FLtbx5b_rev* 5′ TCAACTCCCCCCACACCAGTTG 3′ were used to generate full-length *tbx5b*. Resulting products were cloned into the pGEM T-easy vector (Promega) and further transferred into the *pCS2+* vector to obtain mRNAs. Eighty picograms of mRNA with the corresponding MO (3 ng) were co-injected into one-cell stage embryos and heart laterality assessed at 26 hpf by means of *myl7* expression.

### 4.5. Whole mount *in situ* hybridizations

The antisense RNA probes used were: *myl7* [44], *lefty2* (kindly provided by N. Mercader), *efnb2a* (kindly provided by J. Terriente), *ephB2* (kindly provided by R. Dorsky), *tbx5a* [16], *fgf24* [36], *fgf10a* (obtained by RT-PCR on RNA from 24 hpf with the primers *fwd* 5′ ATGGAAAGTGACTAAGGGTGC 3′ and *rev* 5′ CTACACGATAGGAATGGGGAG 3′) and *pea3* (obtained by RT-PCR with the primers *fwd* 5′ AGAAAGAGCCGCAGAGTCCC 3′ *rev* 5′ TCCTGTTTGACCATCATATGGG 3′).

Chromogenic whole mount *in situ* hybridizations were carried out as described by Albalat *et al.* [16]. Embryos were observed in an OLYMPUS MVX10 macroscope and photographed with the OLYMPUS Cell^D^ software. Fluorescent whole mount *in situs* were carried out as described by Brend *et al.* [45]. Embryos were embedded in 1% low melting agarose (Sigma) dissolved in PBS and observed in a Leica SP2 confocal microscope. Acquired images are projections of z-stacks.

### 4.6. Quantification of retinal phenotypes

The extent of *efnb2a* and *ephB2* expression domain was quantified by setting an imaginary hinge in the centre of the lens. The total angle of expression was sub-divided into nasal versus temporal by considering the choroid fissure as the ventral-most point. The Kruskal–Wallis test was used to assess statistical differences among experimental conditions.

### 4.7. Immunofluorescence

*ath5:GFP* 48 hpf embryos were fixed in 4% paraformaldehyde (PFA) at 4°C, washed with PBST (0.5% Triton), digested with 10 mg ml^−1^ proteinase K for 40 min and post-fixed in 4% PFA for 20 min. After PBST washes, embryos were blocked with 1% BSA and an anti-GFP antibody (Invitrogen, 1 : 600) was subsequently left overnight at 4°C. The antibody was washed out with 1% BSA washes before adding the secondary antibody (anti-rabbit Alexa488 1 : 200, Molecular Probes) and left overnight at 4°C. Secondary antibody washes were performed with PBST. The acquired images are projections of z-stacks taken with a Leica SP2 confocal microscope**.**

## Supplementary Material

Electronic Supplementary Material figures

## References

[1] Begemann G Ingham PW 2000 Developmental regulation of Tbx5 in zebrafish embryogenesis. Mech. Dev. 90, 299–304. (doi:10.1016/S0925-4773(99)00246-4) 10640716 10.1016/s0925-4773(99)00246-4

[2] Chapman DL 1996 Expression of the T-box family genes, *Tbx1–Tbx5*, during early mouse development. Dev. Dyn. 206, 379–390. (doi:10.1002/(SICI)1097-0177(199608)206:4<379::AID-AJA4>3.0.CO;2-F) 8853987 10.1002/(SICI)1097-0177(199608)206:4<379::AID-AJA4>3.0.CO;2-F

[3] Gibson-Brown JJ IAgulnik S Silver LM Papaioannou VE 1998 Expression of T-box genes *Tbx1–Tbx5* during chick organogenesis. Mech. Dev. 74, 165–169. (doi:10.1016/S0925-4773(98)00056-2) 9651516 10.1016/s0925-4773(98)00056-2

[4] Basson CT 1997 Mutations in human TBX5 [corrected] cause limb and cardiac malformation in Holt–Oram syndrome. Nat. Genet. 15, 30–35. (doi:10.1038/ng0197-30) 8988165 10.1038/ng0197-30

[5] Li QY 1997 Holt–Oram syndrome is caused by mutations in TBX5, a member of the *Brachyury* (*T*) gene family. Nat. Genet. 15, 21–29. (doi:10.1038/ng0197-21) 8988164 10.1038/ng0197-21

[6] Agarwal P Wylie JN Galceran J Arkhitko O Li C Deng C Grosschedl R Bruneau BG 2003 *Tbx5* is essential for forelimb bud initiation following patterning of the limb field in the mouse embryo. Development 130, 623–633. (doi:10.1242/dev.00191) 12490567 10.1242/dev.00191

[7] Bruneau BG 2001 A murine model of Holt–Oram syndrome defines roles of the T-box transcription factor Tbx5 in cardiogenesis and disease. Cell 106, 709–721. (doi:10.1016/S0092-8674(01)00493-7) 11572777 10.1016/s0092-8674(01)00493-7

[8] Rallis C Bruneau BG Del Buono J Seidman CE Seidman JG Nissim S Tabin CJ Logan MP 2003 Tbx5 is required for forelimb bud formation and continued outgrowth. Development 130, 2741–2751. (doi:10.1242/dev.00473) 12736217 10.1242/dev.00473

[9] Koshiba-Takeuchi K 2000 Tbx5 and the retinotectum projection. Science 287, 134–137. (doi:10.1126/science.287.5450.134) 10615048 10.1126/science.287.5450.134

[10] Mercader N 2007 Early steps of paired fin development in zebrafish compared with tetrapod limb development. Dev. Growth Differ. 49, 421–437. (doi:10.1111/j.1440-169X.2007.00942.x) 17587327 10.1111/j.1440-169X.2007.00942.x

[11] Glickman NS Yelon D 2002 Cardiac development in zebrafish: coordination of form and function. Semin. Cell Dev. Biol. 13, 507–513. (doi:10.1016/S1084952102001040) 12468254 10.1016/s1084952102001040

[12] Bakkers J 2011 Zebrafish as a model to study cardiac development and human cardiac disease. Cardiovasc. Res. 91, 279–288. (doi:10.1093/cvr/cvr098) 21602174 10.1093/cvr/cvr098 PMC3125074

[13] Chow RL Lang RA 2001 Early eye development in vertebrates. Annu. Rev. Cell Dev. Biol. 17, 255–296. (doi:10.1146/annurev.cellbio.17.1.255) 11687490 10.1146/annurev.cellbio.17.1.255

[14] Garrity DM Childs S Fishman MC 2002 The heartstrings mutation in zebrafish causes heart/fin Tbx5 deficiency syndrome. Development 129, 4635–4645. 12223419 10.1242/dev.129.19.4635

[15] Ahn DG Kourakis MJ Rohde LA Silver LM Ho RK 2002 T-box gene *tbx5* is essential for formation of the pectoral limb bud. Nature 417, 754–758. (doi:10.1038/nature00814) 12066188 10.1038/nature00814

[16] Albalat R Baquero M Minguillon C 2010 Identification and characterisation of the developmental expression pattern of *tbx5b*, a novel *tbx5* gene in zebrafish. Gene Expr. Patterns 10, 24–30. (doi:10.1016/j.gep.2009.11.003) 19925885 10.1016/j.gep.2009.11.003

[17] Parrie LE Renfrew EM Wal AV Mueller RL Garrity DM 2013 Zebrafish *tbx5* paralogs demonstrate independent essential requirements in cardiac and pectoral fin development. Dev. Dyn. 242, 485–502. (doi:10.1002/dvdy.23953) 23441045 10.1002/dvdy.23953

[18] Chen JN van Eeden FJ Warren KS Chin A Nusslein-Volhard C Haffter P Fishman MC 1997 Left–right pattern of cardiac BMP4 may drive asymmetry of the heart in zebrafish. Development 124, 4373–4382. 9334285 10.1242/dev.124.21.4373

[19] Veerkamp J Rudolph F Cseresnyes Z Priller F Otten C Renz M Schaefer L Abdelilah-Seyfried S 2013 Unilateral dampening of Bmp activity by nodal generates cardiac left–right asymmetry. Dev. Cell 24, 660–667. (doi:10.1016/j.devcel.2013.01.026) 23499359 10.1016/j.devcel.2013.01.026

[20] Lenhart KF Holtzman NG Williams JR Burdine RD 2013 Integration of nodal and BMP signals in the heart requires FoxH1 to create left–right differences in cell migration rates that direct cardiac asymmetry. PLoS Genet. 9, e1003109 (doi:10.1371/journal.pgen.1003109) 23358434 10.1371/journal.pgen.1003109 PMC3554567

[21] Plageman TF Jr Yutzey KE 2006 Microarray analysis of Tbx5-induced genes expressed in the developing heart. Dev. Dyn. 235, 2868–2880. (doi:10.1002/dvdy.20923) 16894625 10.1002/dvdy.20923

[22] Gruenauer-Kloevekorn C Reichel MB Duncker GI Froster UG 2005 Molecular genetic and ocular findings in patients with Holt-Oram syndrome. Ophthalmic Genet. 26, 1–8. (doi:10.1080/13816810590918073) 15823919 10.1080/13816810590918073

[23] French CR Erickson T French DV Pilgrim DB Waskiewicz AJ 2009 Gdf6a is required for the initiation of dorsal-ventral retinal patterning and lens development. Dev. Biol. 333, 37–47. (doi:10.1016/j.ydbio.2009.06.018) 19545559 10.1016/j.ydbio.2009.06.018

[24] Mann F Ray S Harris W Holt C 2002 Topographic mapping in dorsoventral axis of the *Xenopus* retinotectal system depends on signaling through ephrin-B ligands. Neuron 35, 461–473. (doi:10.1016/S0896-6273(02)00786-9) 12165469 10.1016/s0896-6273(02)00786-9

[25] Hindges R McLaughlin T Genoud N Henkemeyer M O'Leary D 2002 EphB forward signaling controls directional branch extension and arborization required for dorsal–ventral retinotopic mapping. Neuron 35, 475–487. (doi:10.1016/S0896-6273(02)00799-7) 12165470 10.1016/s0896-6273(02)00799-7

[26] Erickson T French CR Waskiewicz AJ 2010 Meis1 specifies positional information in the retina and tectum to organize the zebrafish visual system. Neural Dev. 5, 22 (doi:10.1186/1749-8104-5-22) 20809932 10.1186/1749-8104-5-22 PMC2939508

[27] Greulich F Rudat C Kispert A 2011 Mechanisms of T-box gene function in the developing heart. Cardiovasc. Res. 91, 212–222. (doi:10.1093/cvr/cvr112) 21498422 10.1093/cvr/cvr112

[28] Naiche LA Harrelson Z Kelly RG Papaioannou VE 2005 T-box genes in vertebrate development. Annu. Rev. Genet. 39, 219–239. (doi:10.1146/annurev.genet.39.073003.105925) 16285859 10.1146/annurev.genet.39.073003.105925

[29] Behesti H Holt JK Sowden JC 2006 The level of BMP4 signaling is critical for the regulation of distinct T-box gene expression domains and growth along the dorso-ventral axis of the optic cup. BMC Dev. Biol. 6, 62 (doi:10.1186/1471-213X-6-62) 17173667 10.1186/1471-213X-6-62 PMC1764729

[30] Gross JM Dowling JE 2005 *Tbx2b* is essential for neuronal differentiation along the dorsal/ventral axis of the zebrafish retina. Proc. Natl Acad. Sci. USA 102, 4371–4376. (doi:10.1073/pnas.0501061102) 15755805 10.1073/pnas.0501061102 PMC555474

[31] Kruse-Bend R Rosenthal J Quist TS Veien ES Fuhrmann S Dorsky RI Chien CB 2012 Extraocular ectoderm triggers dorsal retinal fate during optic vesicle evagination in zebrafish. Dev. Biol. 371, 57–65. (doi:10.1016/j.ydbio.2012.08.004) 22921921 10.1016/j.ydbio.2012.08.004 PMC3455121

[32] Masai I 2003 N-cadherin mediates retinal lamination, maintenance of forebrain compartments and patterning of retinal neurites. Development 130, 2479–2494. (doi:10.1242/dev.00465) 12702661 10.1242/dev.00465

[33] Gyda M Wolman M Lorent K Granato M 2012 The tumor suppressor gene retinoblastoma-1 is required for retinotectal development and visual function in zebrafish. PLoS Genet. 8, e1003106 (doi:10.1371/journal.pgen.1003106) 23209449 10.1371/journal.pgen.1003106 PMC3510048

[34] Vance KW Shaw HM Rodriguez M Ott S Goding CR 2010 The retinoblastoma protein modulates Tbx2 functional specificity. Mol. Biol. Cell 21, 2770–2779. (doi:10.1091/mbc.E09-12-1029) 20534814 10.1091/mbc.E09-12-1029 PMC2912361

[35] Harvey SA Logan MP 2006 *sall4* acts downstream of *tbx5* and is required for pectoral fin outgrowth. Development 133, 1165–1173. (doi:10.1242/dev.02259) 16501170 10.1242/dev.02259

[36] Fischer S Draper BW Neumann CJ 2003 The zebrafish *fgf24* mutant identifies an additional level of Fgf signaling involved in vertebrate forelimb initiation. Development 130, 3515–3524. (doi:10.1242/dev.00537) 12810598 10.1242/dev.00537

[37] Raible F Brand M 2001 Tight transcriptional control of the ETS domain factors Erm and Pea3 by Fgf signaling during early zebrafish development. Mech. Dev. 107, 105–117. (doi:10.1016/S0925-4773(01)00456-7) 11520667 10.1016/s0925-4773(01)00456-7

[38] Chiavacci E Dolfi L Verduci L Meghini F Gestri G Evangelista AM Wilson SW Cremisi F Pitto L 2012 MicroRNA 218 mediates the effects of *Tbx5a* over-expression on zebrafish heart development. PLoS ONE 7, e50536 (doi:10.1371/journal.pone.0050536) 23226307 10.1371/journal.pone.0050536 PMC3511548

[39] McDermott DA Hatcher CJ Basson CT 2008 Atrial fibrillation and other clinical manifestations of altered TBX5 dosage in typical Holt–Oram syndrome. Circ. Res. 103, e96 (doi:10.1161/CIRCRESAHA.108.181834) 18818409 10.1161/CIRCRESAHA.108.181834 PMC2740619

[40] Mori AD 2006 Tbx5-dependent rheostatic control of cardiac gene expression and morphogenesis. Dev. Biol. 297, 566–586. (doi:10.1016/j.ydbio.2006.05.023) 16870172 10.1016/j.ydbio.2006.05.023

[41] Takeuchi JK 2011 Chromatin remodelling complex dosage modulates transcription factor function in heart development. Nat. Commun. 2, 187 (doi:10.1038/ncomms1187) 21304516 10.1038/ncomms1187 PMC3096875

[42] Westerfield M 2000 A guide for the laboratory use of zebrafish (Danio rerio), 4th edn Eugene, OR: University of Oregon Press.

[43] Kimmel CB Ballard WW Kimmel SR Ullmann B Schilling TF 1995 Stages of embryonic development of the zebrafish. Dev. Dyn. 203, 253–310. (doi:10.1002/aja.1002030302) 8589427 10.1002/aja.1002030302

[44] Yelon D Horne SA Stainier DY 1999 Restricted expression of cardiac myosin genes reveals regulated aspects of heart tube assembly in zebrafish. Dev. Biol. 214, 23–37. (doi:10.1006/dbio.1999.9406) 10491254 10.1006/dbio.1999.9406

[45] Brend T Holley SA 2009 Zebrafish whole mount high-resolution double fluorescent *in situ* hybridization. J. Vis. Exp. 25, e1229 (doi:10.3791/1229) 10.3791/1229 PMC2789764 19322135

