# Supplementary material for: Distinct tissue-specific requirements for the zebrafish tbx5 genes during heart, retina and pectoral fin development
Source: Open Biol. 2014 Apr 23;4(4):140014. doi: 10.1098/rsob.140014 (PMC4043114; doi:10.1098/rsob.140014)
Supplement: Electronic Supplementary Material figures [file rsob140014supp1.pdf]

ELECTRONIC SUPPLEMENTARY MATERIAL FIGURE LEGENDS

Supplementary Figure S1. Functionality and efficiency of the MOs used.

(a-a') EGFP expression is observed after injection of 100 pg of *tbx5a-EGFP* mRNA into 1-cell stage embryos. **(b-b')** Co-injection with *tbx5a* MO caused the disappearance of EGFP signal. **(c-c')** Injection of 100 pg of *tbx5b-EGFP* mRNA caused detectable expression of EGFP in 24hpf embryos. **(d-d')** Chimeric *tbx5b* mRNA-driven EGFP expression is abolished by co-injection with the *tbx5b(UTR)* MO. **(e)** RT-PCR from RNA extracted from injected embryos with a *control* MO or a *tbx5b(SP)* MO showing the expected 215 bp (spliced) band in control embryos in comparison to the 791bp (unspliced) bands in *tbx5b(SP)* MO injected embryos.

Supplementary Figure S2. *efnb2a* expression analyses.

(a) Quantification of the total extent of *efnb2a* expression by setting an imaginary hinge in the centre of the lens. **(b)** Dorso-nasal and **(c)** dorso-temporal angles measured by setting the dorsal-most point as the point lying dorsal to the ventrally-located choroid fissure. A Kruskal-Wallis test was used to determine statistical differences among experimental conditions (*P<0.05, **P<0.001).

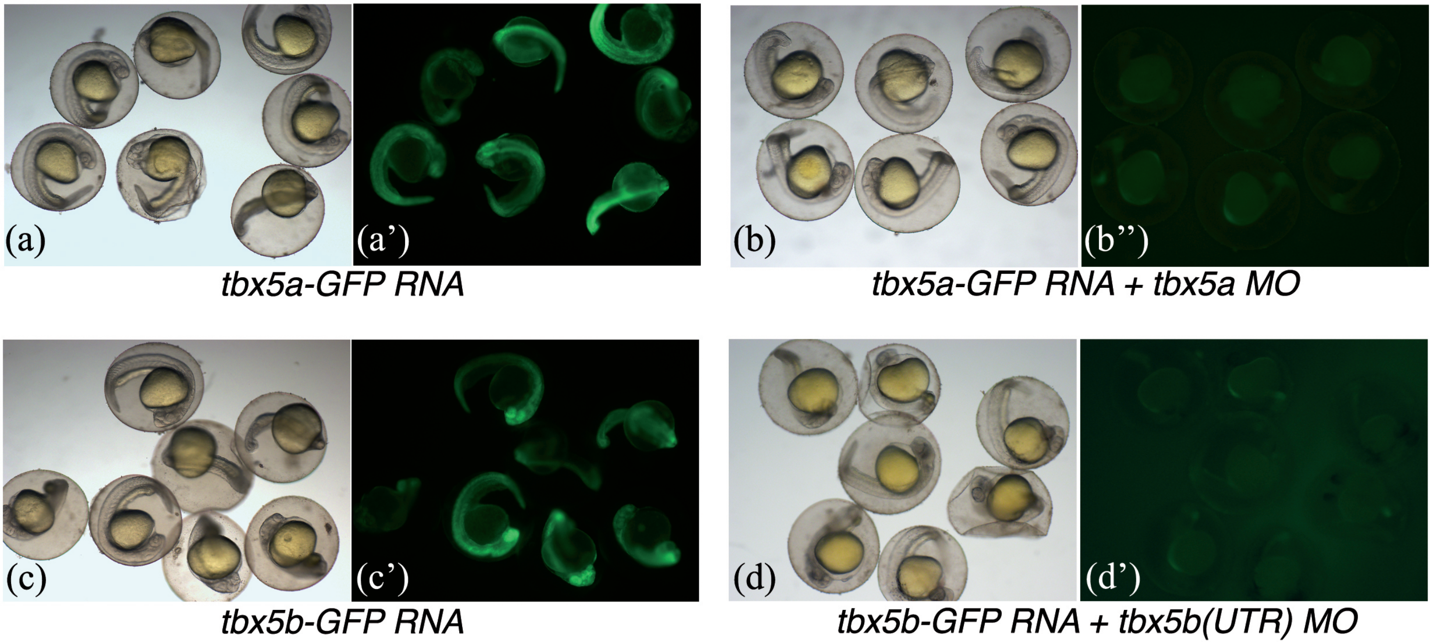

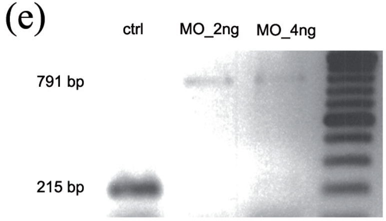

Pi-Roig, electronic supplementary material Figure S1

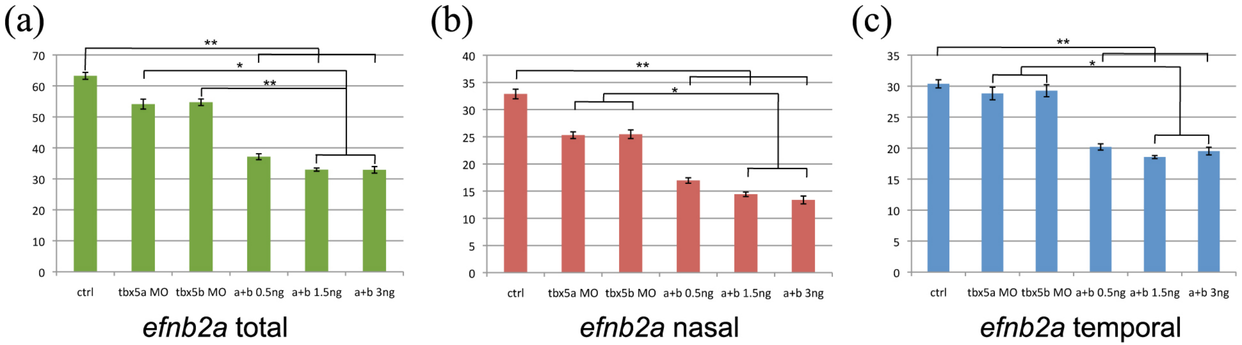

Pi-Roig, electronic supplementary material Figure S2
